# Persistent Childhood and Adolescent Anxiety and Risk for Psychosis: A Longitudinal Birth Cohort Study

**DOI:** 10.1016/j.biopsych.2021.12.003

**Published:** 2022-08-15

**Authors:** Isabel Morales-Muñoz, Edward R. Palmer, Steven Marwaha, Pavan K. Mallikarjun, Rachel Upthegrove

**Affiliations:** a Institute for Mental Health, School of Psychology, University of Birmingham, Birmingham, United Kingdom; b Specialist Mood Disorders Clinic, Zinnia Centre, Birmingham, United Kingdom; c Barberry National Centre for Mental Health, Birmingham, United Kingdom; d Birmingham and Solihull Mental Health Foundation Trust, Birmingham, United Kingdom; e Early Intervention Service, Birmingham Women’s and Children’s NHS Trust, Birmingham, United Kingdom; f Department of Public Health Solutions, Finnish Institute for Health and Welfare, Helsinki, Finland

**Keywords:** ALSPAC, Anxiety, C-reactive protein, Inflammation, Psychosis, Trajectories

## Abstract

**Background:**

Persistent anxiety in childhood and adolescence could represent a novel treatment target for psychosis, potentially targeting activation of stress pathways and secondary nonresolving inflammatory response. Here, we examined the association between persistent anxiety through childhood and adolescence with individuals with psychotic experiences (PEs) or who met criteria for psychotic disorder (PD) at age 24 years. We also investigated whether C-reactive protein mediated any association.

**Methods:**

Data from the Avon Longitudinal Study of Parents and Children (ALSPAC) were available in 8242 children at age 8 years, 7658 at age 10 years, 6906 at age 13 years, and 3889 at age 24 years. The Development and Well-Being Assessment was administered to capture child and adolescent anxiety. We created a composite score of generalized anxiety at ages 8, 10, and 13. PEs and PD were assessed at age 24, derived from the Psychosis-like Symptoms Interview. The mean of C-reactive protein at ages 9 and 15 years was used as a mediator.

**Results:**

Individuals with persistent high levels of anxiety were more likely to develop PEs (odds ratio 2.02, 95% CI 1.26–3.23, *p* = .003) and PD at age 24 (odds ratio 4.23, 95% CI 2.27–7.88, *p* < .001). The mean of C-reactive protein at ages 9 and 15 mediated the associations of persistent anxiety with PEs (bias-corrected estimate −0.001, *p* = .013) and PD (bias-corrected estimate 0.001, *p* = .003).

**Conclusions:**

Persistent high levels of anxiety through childhood and adolescence could be a risk factor for psychosis. Persistent anxiety is potentially related to subsequent psychosis via activation of stress hormones and nonresolving inflammation. These results contribute to the potential for preventive interventions in psychosis, with the novel target of early anxiety.

SEE COMMENTARY ON PAGE e19

Psychosis is a heterogeneous illness with several risk and protective factors (1). Psychotic disorders (PDs) are among the leading causes of disability (2), with a mean incidence of 31.7 per 100,000 people in England (3) and a 12-month prevalence of 1.1% among the U.S. population (4). However, the precise etiology of psychosis remains to be determined (5). Evidence suggests that this involves genetic and environmental risk factors, along with an interaction between the two (6). Well-recognized environmental factors include deprivation, childhood trauma, and minority status (7), which are stressful exposures in early development. Further, individuals who develop psychosis are more likely to experience socioemotional and behavioral problems in childhood (8). However, whether childhood anxiety is specifically associated with subsequent psychosis is still underinvestigated.

Anxiety may have a role in the development of specific psychopathology relevant to psychosis, such as hypervigilance and increased threat to self-network leading to persecutory delusions (9), or as a driving factor for misinterpreting anomalous experiences leading to hallucinations (10,11). After the onset of psychosis, anxiety is common, with prevalence rates for anxiety disorders in psychosis ranging from 42% to 74% (12,13). However, existing studies examining the longitudinal relationship between anxiety and psychosis are scarce, and the results are inconsistent. For instance, associations between changes in depression/anxiety and coexisting psychotic-like experiences over 1 year in adolescence have been reported (14). Further, anxiety was a strong predictor of paranoid thinking at 18-month follow-up in 16- to 74-year-old individuals (15), and having panic attacks precedes psychotic symptoms at ages 18 and 21 (16). To date, previous studies have investigated anxiety at a single time point and in adulthood rather than investigating the continued influence of anxiety in childhood and adolescence. The period of childhood and adolescence is the core risk phase for developing anxiety disorders (17), and anxiety at these ages is a risk factor for general mental disorders in later adolescence and adulthood (18, 19, 20). However, whether persistent high levels of anxiety might be associated with risk of psychosis in the longer term is currently unknown.

If persistent childhood and adolescent anxiety is part of the pathway to psychosis, this may be related to a chronic activation of acute-phase proteins and nonresolving inflammation. C-reactive protein (CRP) may increase proinflammatory cytokines, which could have a direct impact on microglial and astrocytic function that is linked to brain change (21). More specifically, recent hypotheses are emerging suggesting that astrocytic reactivity may reflect impaired restraint by hypofunctional regulatory T cells, leading to subsequent structural and functional brain changes in schizophrenia (22). Further, there is some indication of association between anxiety and higher CRP levels (23, 24, 25, 26), with previous evidence reporting that anxiety symptoms alone increase the probability for elevated CRP levels (23). Recent meta-analyses have also reported a high prevalence of elevated CRP levels in schizophrenia (27,28), and previous research indicates an association between higher CRP levels in adolescence and schizophrenia at follow-up until age 27 years (29). Therefore, evidence exists of an association of CRP with both anxiety and psychotic outcomes. While this may indicate a common inflammatory cause of both anxiety and psychosis (30), an alternative hypothesis might be that chronic anxiety potentiates stress pathways, with downstream impact on risk for psychosis, and as such CRP could potentially mediate the association between anxiety and psychosis.

Understanding the nature of the associations between anxiety in childhood and adolescence with subsequent psychosis would improve knowledge of potential underlying mechanisms of psychosis and provide novel treatment targets. To date, no studies have examined the prospective associations between persistent anxiety across childhood and adolescence and psychosis in adulthood. Further, there is no previous research investigating the mediating role of inflammatory markers in this association. Here, we examined the associations between persistent anxiety across childhood and adolescence with psychosis at age 24 years. Additionally, we investigated whether CRP measured at ages 9 and 15 years mediated these associations. We hypothesized that persistent high levels of anxiety would be a risk factor for psychosis in young adulthood and that CRP levels would mediate these associations.

## Methods and Materials

### Participants

The Avon Longitudinal Study of Parents and Children (ALSPAC) is a birth cohort study in the United Kingdom examining the determinants of development, health, and disease during childhood and beyond (31,32). The ALSPAC study website contains details of all the data available (http://www.bristol.ac.uk/alspac/researchers/our-data/). The initial number of pregnancies enrolled was 14,541. Further details of this cohort are described in the Supplement. Ethical approval was obtained from the ALSPAC Law and Ethics Committee and local research ethics committees. Informed consent was obtained from participants following the recommendations of the ALSPAC Ethics and Law Committee at the time.

### Measures

#### Persistent Anxiety Across Childhood and Adolescence

The Development and Well-Being Assessment (DAWBA) (33) was administered as a parent-report questionnaire to capture child and adolescent psychopathology that corresponds with ICD-10 and DSM-IV criteria. Full details of the DAWBA are provided in the Supplement. We used the generalized anxiety dimension at ages 8, 10, and 13 years as a consistent measure of childhood and early adolescence anxiety, unlikely to be confounded by early symptoms of prodromal psychosis, which typically emerges later in adolescence or early adulthood (34). The generalized anxiety dimension comprises two scores: 1) the generalized anxieties total score, representing the cognitive dimension (e.g. “child worries about disasters,” “child worries about health”); and 2) the generalized anxieties symptoms score, representing the symptomatology dimension (e.g., “child’s general anxieties lead to restlessness,” “child’s general anxieties lead to concentration problems”). We created a composite score of anxiety: (generalized anxieties + generalized anxieties symptoms)/2. We used this composite score to allow capture of two dimensions of anxiety (cognitive and symptomatology) rather than only one (see Table 1 for descriptive values for the variables of interest of this study, including the DAWBA composite score of anxiety).

**Table 1:** Descriptive Values of Sociodemographic and Health-Related Variables, Anxiety Measures, and Psychotic Disorder for the Study Sample

	8 Years Old		10 Years Old		13 Years Old		24 Years Old	
	*n/n* or Mean	%/% or SD	*n/n* or Mean	%/% or SD	*n/n* or Mean	%/% or SD	*n/n* or Mean	%/% or SD
Sociodemographic Factors								
Sex, females/males	4007/4235	48.6%/51.4%	3869/3938	49.6%/50.4%	3444/3462	49.9%/50.1%	2429/1458	62.5%/37.5%
Ethnicity, non-White/White	145/7808	1.8%/98.2%	125/7106	1.7%/98.3%	104/6323	1.6%/98.4%	78/3405	2.2%/97.8%
Birth weight, kg	3.42	0.55	3.42	0.54	3.43	0.54	3.41	0.53
FAI, total score	4.06	4.10	3.93	4.01	3.86	4.01	3.61	3.84
Maternal age at childbirth, years	28.43	4.82	29.03	4.57	28.04	4.68	29.45	4.56
Gestational age, weeks	39.07	2.39	39.41	1.85	39.44	2.31	39.49	1.80
Clinical Scores								
Meeting criteria of PD at 24 years old, Yes/No	–	–	–	–	–	–	47/3842	1.2%/98.8%
PEs at 24 years, Yes/No	–	–	–	–	–	–	120/3842	3.1%/96.9%
DAWBA anxiety composite score	1.16	1.59	2.37	1.61	2.40	1.71	–	–

	9 Years Old		15 Years Old					

Mean (SD)	Range	Mean (SD)	Range				

Inflammatory Markers								
CRP, mg/L	0.80 (2.88)	67.43	1.26 (3.97)	72.48	–	–	–	–
CRP *z*-transformed scores	0.00 (1.06)	24.76	0.00 (1.05)	19.15	–	–	–	–
IL-6	1.29 (1.59)	20.04	–	–	–	–	–	–
IL-6 *z*-transformed scores	0.00 (1.00)	12.60	–	–	–	–	–	–

	*n* (%)	*n* (%)	*n* (%)	*n* (%)				

Substance Use								
Cannabis use at 15 years old	–	–	1347 (25.3%)	3968 (74.7%)	–	–	–	–

CRP, C-reactive protein; DAWBA, Development and Well-Being Assessment; FAI, Family Adversity Index; IL-6, interleukin 6; PD, psychotic disorder; PEs, psychotic experiences.

#### Psychotic Outcomes at 24 Years

Psychotic experiences (PEs) were identified through the semistructured Psychosis-like Symptom Interview (35). PEs occurring in the past 6 months included the three main positive symptom domains: hallucinations, delusions, and thought interference. Interviewers rated PEs as not present, suspected, or definitely present. Cases of PEs were defined as individuals with definite PEs.

Following previous research (36,37), we identified PD (i.e., meeting criteria for PD) at 24 years, a more restricted phenotype, which was defined as follows: 1) being rated as having definite PEs not attributable to sleep or fever; 2) having recurred regularly over the previous 6 months; 3) being reported as very distressing or having very negative impact on their social/occupational functioning. We selected the psychotic outcomes at age 24 to give a clear 11-year time frame between the last measure of anxiety (age 13) and subsequent psychosis.

#### Inflammatory Markers at 9 and 15 Years

Blood samples were collected from nonfasting participants during clinic assessment at age 9 around the same time of the day. At age 15, blood was drawn while fasting at a largely consistent time of day (i.e., at least 6 hours), limiting potential for diurnal effects on traits such as inflammatory markers (38). Samples were immediately spun, frozen, and stored at −80 °C. There was no evidence of freeze-thaw cycles during storage. High-sensitivity CRP was measured at one time point at the same laboratory by automated particle-enhanced immunoturbidimetric assay (Roche UK). Additionally, interleukin 6 (IL-6) was measured by single enzyme-linked immunosorbent assay (R&D Systems). All assay coefficients of variation were <5%. CRP was available at ages 9 and 15, and this was the main inflammatory marker selected for this study owing to the availability of this marker at two time points, which allowed us to treat this variable as a mediator. IL-6 was available only at age 9, and thus it could not be treated as a mediator, but only as a contributing factor. Therefore, this was not included in the main analyses of this study; results for IL-6 are, however, given in Figure S1.

#### Confounders

Multiple family risk factors were assessed using the Family Adversity Index (FAI) during pregnancy (long index), at 2 years (long index), and at 4 years (short index). FAI includes early parenthood, housing and family conditions, maternal education, financial difficulties, parents’ relationship, maternal psychopathology, parents’ substance abuse, partner support, and social network. Points were summed at each time point for a total FAI score. We included this variable as a confounder, as early adversity is a well-established risk factor for poor mental health (39). We also controlled for cannabis use at age 15, as adolescent cannabis use is related to anxiety and is a risk factor for PD and PEs (40,41).

Finally, relevant socioeconomic factors selected as covariates were child’s sex, gestational age, and ethnicity and maternal age when the child was born. Child’s sex, gestational age, ethnicity and maternal age at childbirth were selected because of their impact on psychosis and anxiety (42).

### Statistical Analysis

First, we conducted latent class growth analyses using Mplus (version 8; Muthén & Muthén) to potentially identify differing levels of anxiety symptoms across childhood and adolescence. The indicator variables were DAWBA composite score of anxiety at ages 8, 10, and 13 years. Several models were fitted by increasing the number of classes (43). The best-fitting classification model was chosen according to fit indices (i.e., Bayesian information criteria [BIC] and Vuong-Lo-Mendell-Rubin [VLMR] test) (43). Lower BIC values suggest better model fit. A significant VLMR value suggests that a k-class model fits the data better than a K − 1 class model. Entropy was additionally used to select the best model fit; entropy with values approaching 1 indicates clear delineation of classes. Finally, to decide the optimal class solution, an emphasis was placed on large enough group sizes. Missing values owing to attrition were handled by the full information maximum likelihood estimation method (43).

Second, we investigated the prospective associations between persistent high levels of anxiety, identified by latent class growth analyses, and psychosis at age 24 using SPSS version 25 (IBM Corp). We conducted multinomial logistic regression analyses. The derived latent classes from the latent class growth analyses were included as predictor (with class 1, which referred to persistent low levels of anxiety, as reference) and psychosis at age 24 as the outcome. We included the two psychotic outcomes (i.e., PEs and PD) in separate models. Further, we tested first unadjusted associations, and then we controlled for all the confounders in the adjusted model. To deal with missingness, we conducted logistic regressions to identify significant factors associated with attrition. The individuals associated with attrition at age 24 were more often boys and had younger mothers, shorter gestational age, lower weight at birth, and higher socioeconomic levels (Table S1). Using the variables associated with selective dropout as the factors, we fitted a logistic regression model to determine weights for each individual using the inverse probability of response.

Finally, to examine the potential role of CRP at ages 9 and 15 as a mediator in the association between persistent high levels of anxiety in childhood and adolescence with psychosis at age 24, we conducted path analysis in SPSS Amos 27 (IBM Corp.). Two separate path analyses were conducted for each psychotic outcome (i.e., PEs and PD). CRP levels at each time point (9 and 15 years) were standardized (*z*-transformed), and then we computed the mean score of CRP (*z*-transformed) at 9 and 15 years. Therefore, this new mean score ([CRP9 + CRP15]/2) was included as a mediator in the path analyses. The independent variable was dichotomized: 1 = class referring to persistent high levels of anxiety across time points; 0 = the other classes. We controlled for FAI, sex, birth weight, and cannabis use at age 15. We used bootstrapped bias-corrected 95% confidence intervals and *p* values for assessing the significance of the standardized indirect associations. Missing data were dealt with using the full information maximum likelihood method. Further, additional analyses to examine the potential role of IL-6 at age 9 in the associations between persistent anxiety and psychotic outcomes at age 24 were conducted.

## Results

### Latent Classes of Anxiety

**Figure 1:**
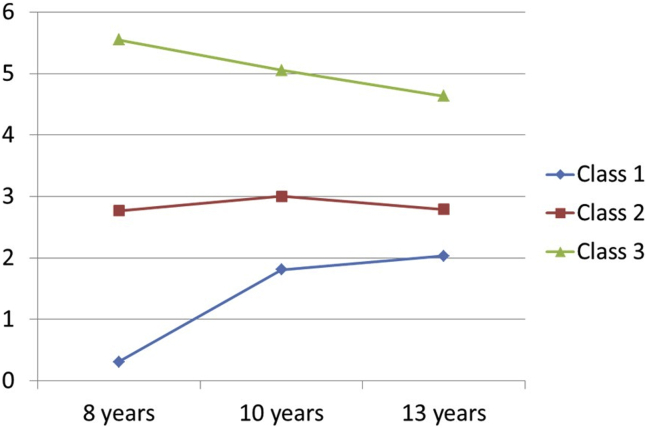
Growth trajectories of anxiety across childhood to adolescence. The latent class growth analyses detected a best model fit for 3 classes. Class 1 (blue line on the bottom) represents individuals with persistent low levels of anxiety across time points. Class 2 (red line in the middle) represents individuals with persistent intermediate levels of anxiety. Class 3 (green line on the top) represents individuals with persistent high levels of anxiety across time points, which was the main focus of this study.

Table 2 shows VLMR, BIC, and entropy for all models assessed (2–6 classes). Overall, a 3-class model offered the best fit. VLMR showed a statistically significant difference for the 2-class, 3-class, and 4-class models. The 5-class model did not offer a significantly better fit than the 4-class model at the *p* < .01 level. Further, BIC decreased with the addition of each class, indicating a better model fit for more classes. This pattern is typically found in large samples (44). However, decreases in BIC became considerably smaller in 3-class compared with 2-class models. Finally, compared with the 4-class model, the 3-class model reported higher entropy value (near 1.0), which refers to classification precision. As this is important when importing classes for further analyses (45), the 3-class model with an entropy of 0.864 was selected. In addition, the 3-class model provided large enough group sizes for each class (class 1 = 6331; class 2 = 1882; class 3 = 469), whereas 2 or 4-class models produced mixed or smaller groups (Table S2). The 3 derived classes of anxiety from the 3-class model are shown in Figure 1. The derived classes from the 2- and 4-class models appear in Table S2. In the 3-class model, class 3 represented persistent high levels of anxiety (5.4% of the sample), class 2 represented persistent moderate levels of anxiety (21.7% of the sample), and class 1 represented persistent low levels of anxiety (72.9% of the sample). Descriptive values for the covariates, psychotic measures, and inflammatory markers for each of the 3 classes appear in Table S3.

**Table 2:** BIC, VLMR Likelihood Test *p* Values, and Entropy for Classes 2–6 of the DAWBA Composite Score of Anxiety

Composite Score of General Anxieties	BIC	VLMR *p* Value	Entropy
2 Classes	47499.749	<.001	0.853
3 Classes	45831.340	.0014	0.864
4 Classes	45640.278	.0007	0.817
5 Classes	43290.281	.0481	0.884
6 Classes	43317.489	.2610	0.833

BIC, Bayesian information criterion; DAWBA, Development and Well-Being Assessment; VLMR, Vuong-Lo-Mendell-Rubin.

### Associations Between Persistent High Levels of Anxiety and Psychosis at 24 Years

The weighted adjusted logistic regression model showed that persistent high levels of anxiety across time points (i.e., class 3) were significantly associated with PEs at age 24 years (odds ratio [OR] 2.02, 95% CI 1.26–3.23, *p* = .003) and PD at age 24 (OR 4.23, 95% CI 2.27–7.88, *p* = .001) (Table 3). To show the consistency and robustness of these findings, logistic regression analyses with other psychosis-related outcomes at age 24 are reported in Tables S4 through S6, where similar trends were found for broader defined psychotic outcomes. Further, to explore the specificity of our findings, logistic regression analyses with other relevant mental disorders (e.g., depression, anxiety, hypomania, substance abuse) in young adulthood were also performed. We found that persistent anxiety was also associated with depression and generalized anxiety at age 24. The ORs of these associations (i.e., 1.99 and 2.15, respectively) were lower than the OR reported in the association between persistent anxiety and PD (i.e., 4.23). Further, persistent high anxiety was not associated with hypomania, phobias, or substance abuse in young adulthood. This supports the specificity of the associations reported here in relation to psychosis at age 24 (see Tables S7–S10 for further details).

**Table 3:** Associations of Latent Classes of Anxiety With Psychotic-like Symptoms and Meeting Criteria of Psychotic Disorder at 24 Years

	Unadjusted Model			Adjusted Model		
	OR	95% CI	*p* Value	OR	95% CI	*p* Value
Psychotic Experiences at 24 Years						
General Anxiety Class 1 (Reference)	–	–	.035	–	–	.011
General Anxiety Class 2	0.908	0.680–1.214	.515	0.989	0.708–1.381	.949
General Anxiety Class 3	1.698^a^	1.096–2.628^a^	.018^a^	2.022^a^	1.265–3.232^a^	.003^a^
Sex	–	–	–	1.344^a^	1.014–1.781^a^	.040^a^
Gestational Age	–	–	–	1.007	0.929–1.091	.868
FAI Total Score	–	–	–	1.003	0.972–1.035	.857
Ethnicity	–	–	–	1.734	0.817–3.681	.152
Maternal Age at Childbirth	–	–	–	1.017	0.986–1.049	.280
Cannabis Use Ever at 15 Years Old	–	–	–	1.673^a^	1.239–2.260^a^	.001^a^
Meeting Criteria of Psychotic Disorder at 24 Years						
General Anxiety Class 1 (Reference)	–	–	.004	–	–	.000
General Anxiety Class 2	1.023	0.651–1.609	.920	1.392	0.809–2.394	.232
General Anxiety Class 3	2.656^a^	1.479–4.767^a^	.001^a^	4.229^a^	2.268–7.885^a^	< .001^a^
Sex	–	–	–	0.659	0.415–1.048	.078
Gestational Age	–	–	–	0.936	0.831–1.055	.279
FAI Total Score	–	–	–	1.038	0.989–1.090	.129
Ethnicity	–	–	–	1.673	0.500–5.589	.403
Maternal Age at Childbirth	–	–	–	1.001	0.952–1.054	.959
Cannabis Use Ever at 15 Years Old	–	–	–	0.847	0.475–1.512	.575

In relation to psychotic experiences at 24 years old, in addition to the significant associations observed between persistent anxiety (class 3) and the outcome, we also found that being a boy and cannabis use at 15 years old both were related to psychotic experiences at 24 years. However, concerning meeting criteria of psychotic disorder at 24 years old, none of the covariates included were associated with the outcome at 24 years old.

FAI, Family Adversity Index; OR, odds ratio.

a Significant value.

### Mediating Effect of Inflammatory Markers

**Figure 2:**
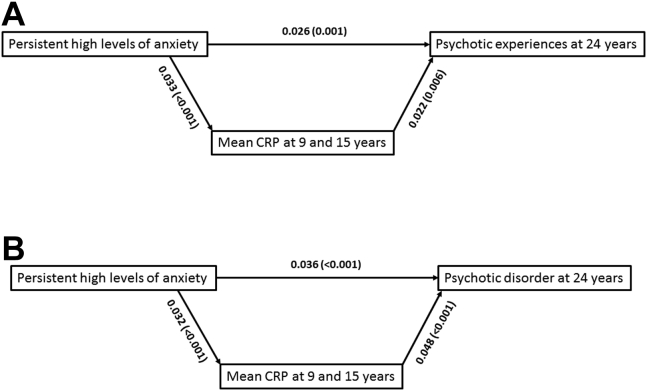
Path diagram showing the main direct associations. Only the direct associations of the independent variable, mediating factor, and dependent variable are shown. Persistent high levels of anxiety represent the independent variable; the mean of C-reactive protein (CRP) levels at ages 9 and 15 represent the mediating factor; and psychotic experiences **(A)** and meeting criteria for psychotic disorder **(B)** at age 24 represent the outcomes. The covariates also included in this path analysis were sex, family adversity, birth weight, and cannabis use at age 15. Significant pathways are indicated by solid arrows.

In examining whether the mean level of CRP at ages 9 and 15 years mediated the association between persistent anxiety and PEs at age 24, path analysis model fit indices indicated good model fit (χ^2^ = 3.26, *p* = 0.66, root mean square error of approximation 0, comparative fit index 1.00). Consistent with the adjusted logistic regression, persistent high levels of anxiety across time points were directly and significantly associated with PEs at age 24 (β = 0.028, *p* < .001). Direct associations are shown in Figure 2A. Further, we observed an indirect effect of the mean of CRP at ages 9 and 15 in the association between exposure and outcome (bias-corrected estimate −0.001, 95% CI −0.002 to 0.000, *p* = .013). The fact that the bias-corrected estimate includes the value 0 indicates certain uncertainty of these results. The mediating role of the mean of CRP at ages 9 and 15 in the association between persistent anxiety and PD at age 24 also demonstrated a good model fit (χ^2^ = 2.80, *p* = .73, root mean square error of approximation 0, comparative fit index 1.00). Persistent anxiety was directly and significantly associated with PD at age 24 (β = 0.040, *p* < .001). Direct associations are shown in Figure 2B. Further, an indirect effect of the mean of CRP at 9 and 15 in the association between exposure and outcome was found (bias-corrected estimate 0.001, 95% CI 0.001–0.002, *p* = .003). Additionally, path analyses with IL-6 at age 9 as mediator are provided in Figure S1. IL-6 at age 9 did not mediate the associations between persistent anxiety and any of the psychotic outcomes.

## Discussion

To our knowledge, this is the first longitudinal study to examine whether persistent high levels of anxiety across childhood and adolescence are associated with PEs and PD at age 24 and to investigate potential inflammatory mechanisms mediating this association. First, we detected a group of individuals characterized by persistent high levels of anxiety across childhood and adolescence. Second, persistent high levels of anxiety were associated with PEs and PD at 24 years. Finally, CRP levels mediated the prospective associations between persistent high levels of anxiety and psychosis at age 24 (i.e., PEs and PD).

We identified 3 different trajectories of anxiety across childhood and adolescence. These were persistent low levels of anxiety, comprising more than 70% of the sample; persistent moderate levels of anxiety, representing approximately 20% of the sample; and persistent high levels of anxiety, representing 5% of the sample. These findings support recent research reporting the presence of 3 classes of individuals with distinct anxiety symptom trajectories in early adolescence (46, 47, 48). It appears that it is possible to identify a group of children and adolescents who experience persistent and high anxiety levels, and these could represent individuals at higher risk for later mental disorders, including psychosis.

To our knowledge, this is the first study to report the associations between persistent high levels of anxiety across childhood and adolescence with psychosis at age 24. These findings contribute novel evidence that supports the role of anxiety in early stages as a precursor of psychosis. Interestingly, we found more robust associations of persistent high levels of anxiety with PD than with PEs. This suggests that persistent anxiety across childhood and adolescent might constitute a better indicator of the development of future formal PD, while PEs, which are far more common in the population than PD (49), constitute a more heterogeneous group. As such, PEs may be associated with a wider range of potential risk factors in young adulthood. This is supported in our study by the fact that none of our covariates were associated with PD, while sex and cannabis use were both associated with PEs. Our findings build on the literature of an association between anxiety and psychosis, previously related to psychotic symptom severity, distress of positive symptoms, and associated prognosis and relapse (50). However, much of this previous work is cross-sectional, or focused on persistence and generation of positive presence of childhood anxiety many years before the onset of psychosis. In one previous large longitudinal study, anxiety at a single time point (age 15 years) was not associated with PD in young adulthood (20). Our study indicates that it is the persistence of high anxiety that might constitute a risk for future psychosis, even after adjusting for multiple important confounders. Therefore, our findings highlight the potential causal role of persistent anxiety in the development of psychosis and potentially support the notion that genetic risk for the disorder may be associated with increased anxiety long before the onset of psychosis (51, 52). Further, our findings indicate that the associations of persistent high levels of anxiety with psychosis at age 24 could be specific to this mental disorder, as no associations with other relevant disorders, such as hypomania, phobias, or substance abuse, were found. However, the potential role of other persistent symptoms in the findings, particularly persistent depression, which is usually comorbid with anxiety (53), could also explain some of our findings.

The mediating role that CRP exerted in the association between persistent high levels of anxiety across childhood and adolescence and both PEs and PD at 24 years is noteworthy. While inflammation in childhood is a risk factor for depression (54), anxiety (55), and psychosis (56), our novel approach was to identify a persistent clinically relevant phenotype (anxiety) and demonstrate relevance for psychosis, with potentially CRP mediating these associations. We found that the mean of CRP levels at ages 9 and 15 mediated the associations between persistent high anxiety and psychotic outcomes at age 24 (i.e., PEs and PD). Persistently high levels of anxiety may lead to increased levels of stress hormones, which would activate a chronic, low-grade inflammatory state (57). Within this context, the CRP plasma levels may indicate nonresolving inflammation, activation of microglia, and subsequent downstream effects on brain structure and function via disordered synaptic pruning affecting crucial neurodevelopmental stages, ultimately leading to psychosis (27,28). Our findings provide new evidence suggesting that persistent high levels of anxiety are associated with psychosis, and this may be mediated by elevated CRP levels. However, activation of stress pathways and inflammation is only one potential explanation of these associations, and further research should disentangle in more detail this potential mechanism, including interrogation of genetic factors and further assessment of other environmental factors. In addition, further interrogation of directionality of association would need to be explored in further observational and experimental medicine studies within inflammatory markers. Finally, we did not find a mediating role of IL-6 at age 9 in any of these associations, which is in contrast to increasing evidence of IL-6 in relation to schizophrenia with anxiety (58). Studies including IL-6 at more than one time point and at later stages are required to further mechanistic understanding of persistent anxiety and psychosis at age 24.

This study has several strengths, including the large population-based sample, the longitudinal design, and the inclusion of meeting criteria for both PD and PEs at age 24. There are also some limitations. First, other potential contributing factors, such as depression, cognition, social interactions, obstetric complications, or factors associated with CRP, such as viral infections, smoking, or body mass index, were left unexplored. Second, our study was unable to use anxiety data in individuals older than 13 years, and thus we cannot comment on a trajectory of future anxiety disorders after this age group. Third, the attrition rate was significant. However, we used procedures to ensure representativeness of our results. Fourth, anxiety identified at age 13 may be part of prodromal symptoms in an early-onset group; however, our premise is that persistence of anxiety was the exposure of interest, and it is unlikely that anxiety at age 9 is conflated with prodromal psychosis. Fifth, only 47 individuals presented with criteria for PD, comprising 1.2% of our sample at age 24. However, this prevalence is representative of the general population. Sixth, we focused on the impact of persistent high anxiety on psychosis, and the effect of other potential anxiety trajectories, such as worsening anxiety, were left unexplored. Future studies should focus on examining also how worsening anxiety might be associated with psychosis. Finally, our results concerning the mediating role of CRP were more robust in relation to PD compared with PEs. For instance, we found a positive indirect effect between persistent anxiety and PD, which was congruent with the positive direct effects observed between exposure, mediator, and outcome. However, in relation to PEs, we found a negative indirect effect, despite direct effects between the three variables being reported. Therefore, our results in relation to PEs should be cautiously interpreted. One possible explanation for the inconsistency of the direction of the indirect effect in PEs could be that other potential mediators would explain the negative indirect effect observed. As previously reported in the discussion, PEs are associated with a wider range of risk factors than PD, which would subsequently impact our findings. Further, the bias-corrected estimate in relation to the indirect effect for PEs contains the value 0, and thus there are limited conclusions that can be drawn. This supports the need for future studies to disentangle other potential mechanisms underlying this association.

In summary, our findings showed that persistent high levels of anxiety across childhood and adolescence are associated with psychosis at age 24. Further, CRP levels at ages 9 and 15 had a mediating role in these prospective associations. These findings suggest that persistent high levels of anxiety predate and could be considered a potentially malleable phenotype for developing psychosis at age 24, which could inform targeted prevention strategies. Moreover, a nonresolving proinflammatory mechanism might partially explain how persistent anxiety might lead to subsequent psychosis and provides further support to the role of inflammation in mechanistic pathways to psychosis. These results could contribute to the design of more personalized and indicated prevention in psychosis, such as early diagnosis and management of adolescent anxiety and possibly novel treatments targeted at inflammation.

## Supplementary Material

Supplementary Material

Key Resources Table
