# Supplementary material for: Persistent Childhood and Adolescent Anxiety and Risk for Psychosis: A Longitudinal Birth Cohort Study
Source: Biol Psychiatry. 2022 Aug 15;92(4):275–82. doi: 10.1016/j.biopsych.2021.12.003 (PMC9302897; doi:10.1016/j.biopsych.2021.12.003)
Supplement: Supplementary Material [file mmc1.pdf]

Persistent Childhood and Adolescence Anxiety and Risk for Psychosis: A Longitudinal Birth Cohort Study

Supplementary Information

Further details of ALSPAC cohort (page 2)

Further details of the DAWBA (page 3)

Table S1. Differences in socio-demographic variables between non-participating and participating subjects in the study (page 4)

Table S2. Number of individuals per class, for each LCGA 2classes model, 3classes-model and 4 classes-model (page 5)

Table S3. Descriptive values for the covariates, psychotic measures and inflammatory markers for each class from the LCGA (page 6)

Table S4. Logistic regression analyses between 3-classes model of general anxiety and Psychotic symptoms at 24 years (page 7)

Table S5. Logistic regression analyses between 3-classes model of general anxiety and Psychotic disorder at 24 years, broad (page 8)

Table S6. Logistic regression analyses between 3-classes model of general anxiety and Psychotic episode at 24 years (page 9)

Table S7. Logistic regression analyses between 3-classes model of general anxiety and depression at 24 years (page 10)

Table S8. Logistic regression analyses between 3-classes model of general anxiety and anxiety at 24 years (page 11)

Table S9. Logistic regression analyses between 3-classes model of general anxiety and hypomania at 24 years (page 12)

Table S10. Logistic regression analyses between 3-classes model of general anxiety and substance abuse at 24 years (page 13)

Fig S1. Path diagram showing the main direct associations between persistent anxiety, IL-6 at 9 years and psychotic outcomes at 24 years (page 14)

Table S11. Descriptive values (Mean and SD) of the inflammatory marker values in persistent anxiety, PE at 24 years and psychotic disorder at 24 (page 15)

Further details of ALSPAC cohort:

Pregnant women resident in Avon, UK with expected dates of delivery 1st April 1991 to 31st December 1992 were invited to take part in the study. The initial number of pregnancies enrolled was 14, 541 (for these at least one questionnaire was returned, or a "Children in Focus" clinic had been attended by 19/07/99). Of these initial pregnancies, there was a total of 14676 foetuses, resulting in 14062 live births and 13988 children who were alive at 1 year of age. When the oldest children were approximately 7 years of age, an attempt was made to bolster the initial sample with eligible cases who had failed to join the study originally. As a result, in our study, as some variables were collected from the age of seven onwards there were data available for more than the 14541 pregnancies mentioned above. Informed consent for the use of data collected via questionnaires and clinics was obtained from participants following the recommendations of the ALSPAC Ethics and Law Committee at the time. Ethical approval was obtained from the ALSPAC Law and Ethics committee and the local research ethics committees.

Further details of the DAWBA:

The DAWBA is a package of interviews, questionnaires and rating techniques designed to generate ICD-10 and DSM-IV or DSM-5 psychiatric diagnoses about 2-17 years old. The DAWBA includes a mix of 'closed'/structured questions and open-ended questions, where respondents describe their difficulties in their own words. The full DAWBA package covers the following diagnoses: Separation anxiety, Specific phobia, Social phobia, Panic disorder/agoraphobia, Post-traumatic stress disorder, Obsessive compulsive disorder, Generalized anxiety disorder, Body dysmorphic disorder, Disruptive mood dysregulation disorder, Major depression, ADHD/hyperkinesis, Oppositional defiant disorder, Conduct disorder, Eating disorders, including anorexia, bulimia and binge eating, Autism spectrum disorders, Tic disorders, including Tourette syndrome, and Bipolar disorders. For each of these disorders, the interview asks about all the symptoms, and other criteria needed for an operationalized diagnosis according to both DSM-IV (American Psychiatric Association, 1994) and the research diagnostic version of ICD-10 (World Health Organisation, 1994). Panic disorder, agoraphobia, autistic disorders, eating disorders, tic disorders, and any other concerns are covered more briefly, with clinical diagnoses of these disorders being correspondingly more dependent on rating the open-ended transcript. The time frame of the interview is the present and the recent past. For many disorders, the ICD-10 and DSM-IV diagnostic criteria stipulate that the symptoms need to have persisted for a specified number of months, e.g. a minimum of 6 months for hyperactivity, oppositional-defiant disorder, and generalized anxiety disorders. In these instances, the relevant section of the DAWBA interview focuses on the child's symptoms over this

stipulated period. The time frame is longest for conduct disorder (since DSM-IV criteria include the number of relevant behaviours displayed over the previous 12 months), and shortest for most of the emotional disorders, where the focus is on the last month, in line with previous recommendations (Shaffer et al., 1996).

Table S1. Differences in socio-demographic variables between non-participating and participating subjects in the study

	Non-participating group in the study		Participating group in the study (at 24 years old)		Non-participating versus participating	
	Mean	*SD*	*Mean*	*SD*	*OR (95% CI)*	*p*
Maternal age when born	27.49	4.99	29.45	4.56	1.08 (1.08, 1.09)	<0.001
Gestational age	38.00	6.22	39.49	1.80	1.09 (1.08, 1.11)	<0.001
Birth weight, grams	3371.23	596.18	3410.33	532.74	1.00 (1.00, 1.00)	0.001
Family Adversity score	4.74	4.47	3.61	3.84	0.936 (0.93, 0.95)	<0.001
	**Non-participating group in the study**		**Participating group in the study**			
	N	*%*	*N*	*%*		
Sex						
Male / Female	6233 / 4919	55.9 / 44.1	1458 / 2429	37.5 / 62.5	0.47 (0.44, 0.51)	<0.001
Ethnicity						
White / Other	8657 / 248	97.2 / 2.8	3405 / 78	97.8 / 2.2	1.251 (0.97, 1.62)	0.089

The individuals associated with attrition at 24 years were more often boys, their mothers were younger when baby was born, the gestational age was shorter, they weighted less at birth, and they had higher socioeconomic levels.

Table S2. Number of individuals per class, for each LCGA 2classes model, 3classes-model and 4 classes-model

	**LCGA 2-classes model**		**LCGA 3-classes model**			**LCGA 4-classes model**			
	Class 1	Class 2	Class 1	Class 2	Class 3	Class 1	Class 2	Class 3	Class 4
N	7335	1347	6331	1882	469	90	1867	6238	487
Percentage	84.5%	15.5%	72.9%	21.7%	5.4%	1.0%	21.5%	71.8%	5.6%

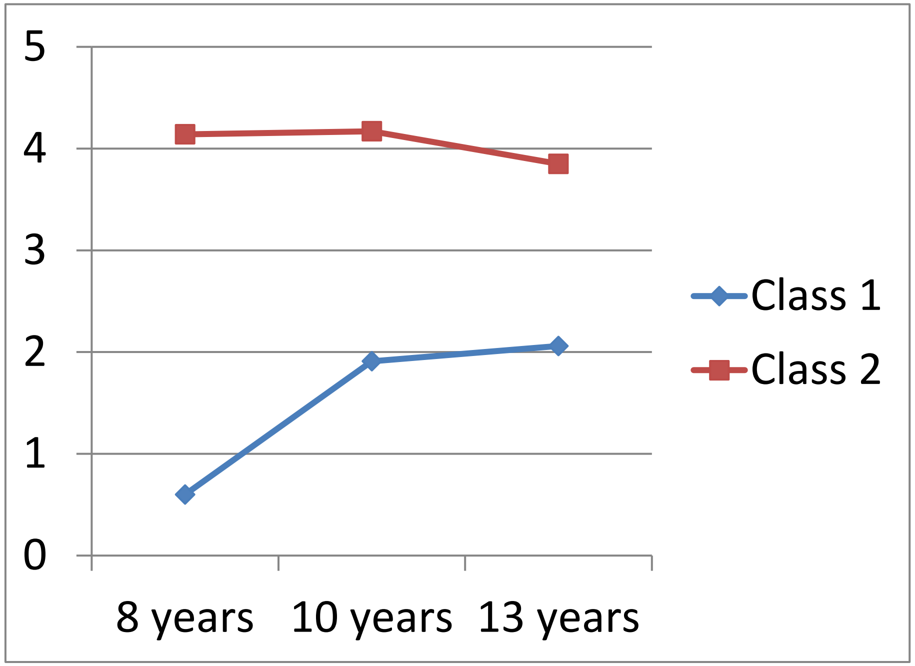

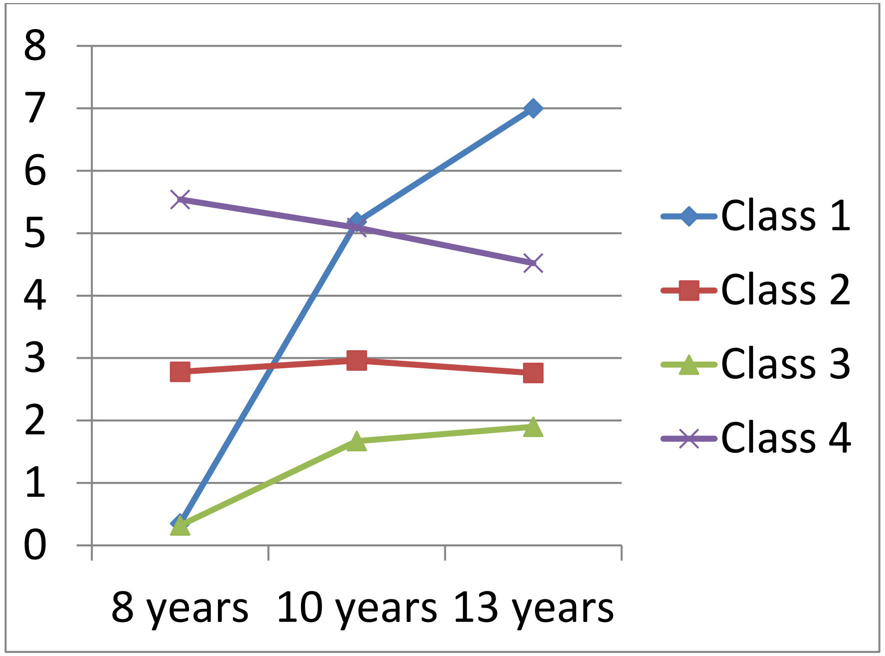

Table S3. Descriptive values for the covariates, psychotic measures and inflammatory markers for each class from the LCGA

	**Class 1**		**Class 2**		**Class 3**	
	Mean	SD	Mean	SD	Mean	SD
Birthweight, gr	3430.28	545.79	3403.80	541.53	3356.90	578.809
FAI score	3.93	4.09	4.32	4.08	5.82	5.03
Gestational age	39.44	1.88	39.47	1.85	39.43	2.00
Maternal age	28.88	4.62	29.04	4.62	28.33	4.79
CRP at 9 years	0.73	2.45	0.86	3.03	0.94	3.40
CRP at 15 years	1.23	3.85	1.21	4.05	1.26	3.50
IL-6 at 9 years	1.26	1.54	1.33	1.64	1.33	1.43

	**Class 1**		**Class 2**		**Class 3**	
	N	%	N	%	N	%
Sex<br>Boys / girls	3251 / 3080	51.4 / 48.6	934 / 948	49.6 / 50.4	236 / 233	50.3 / 49.7
Cannabis use at 15y<br>Yes / No	816 / 2461	24.9 / 75.1	258 / 805	24.3 / 75.7	67 / 189	26.2 / 73.8
Ethnicity<br>White / non-white	5854 / 113	98.1 / 1.9	1773 / 27	98.5 / 1.5	430 / 8	98.2 / 1.8
Psychotic disorder 24y<br>Yes / No	24 / 2248	1.1 / 98.9	10 / 765	1.3 / 98.7	4 / 158	2.5 / 97.5
PEs 24y<br>Yes / No	60 / 2212	2.6 / 97.4	28 / 747	3.6 / 96.4	7 / 155	4.3 / 95.7

Table S4. Logistic regression analyses between 3-classes model of general anxiety and Psychotic symptoms at 24 years

**Psychotic symptoms at 24 years***						
	**Unadjusted model**			**Adjusted model**		
	OR	CI 95%	p	OR	CI 95%	p
General anxiety Class 1 (ref)	---	---	0.021	---	---	0.004
General anxiety Class 2	0.915	0.623 to 1.345	0.652	1.214	0.769 to 1.916	0.406
General anxiety Class 3	**2.047**	**1.200 to 3.491**	**0.009**	**2.770**	**1.528 to 5.022**	**0.001**
Sex	---	---	---	0.822	0.557 to 1.213	0.323
Gestational age	---	---	---	1.004	0.897 to 1.123	0.949
FAI total score	---	---	---	1.035	0.993 to 1.077	0.101
Ethnicity	---	---	---	1.186	0.357 to 3.935	0.781
Maternal age when birth	---	---	---	1.001	0.959 to 1.045	0.948
Cannabis use ever at 15 years	---	---	---	0.800	0.514 to 1.246	0.324

*Psychotic symptoms during the past 6 months, not attributable to sleep or fever, and very distressing or having a very negative impact on their social or occupational functioning

Table S5. Logistic regression analyses between 3-classes model of general anxiety and Psychotic disorder at 24 years, broad

Psychotic disorder at 24 years, broad*						
	**Unadjusted model**			**Adjusted model**		
	OR	CI 95%	p	OR	CI 95%	p
General anxiety Class 1 (ref)	---	---	<0.001	---	---	<0.001
General anxiety Class 2	1.141	0.746 to 1.747	0.543	1.599	0.967 to 2.642	0.067
General anxiety Class 3	**4.037**	**2.475 to 6.583**	**<0.001**	**6.259**	**3.681 to 10.643**	**<0.001**
Sex	---	---	---	0.775	0.508 to 1.181	0.235
Gestational age	---	---	---	0.943	0.845 to 1.053	0.296
FAI total score	---	---	---	1.022	0.975 to 1.070	0.365
Ethnicity	---	---	---	**4.117**	**1.890 to 8.996**	**<0.001**
Maternal age when birth	---	---	---	1.020	0.974 to 1.069	0.401
Cannabis use ever at 15 years	---	---	---	1.192	0.696 to 2.040	0.523

*Psychotic disorder at 24 years, broad, including reduced function as option for impairment.

Table S6. Logistic regression analyses between 3-classes model of general anxiety and Psychotic episode at 24 years

	**Psychotic episode at 24 years***					
	**Unadjusted model**			**Adjusted model**		
	OR	CI 95%	p	OR	CI 95%	p
General anxiety Class 1 (ref)	---	---	0.001	---	---	0.001
General anxiety Class 2	0.921	0.699 to 1.213	0.559	**0.758**	**0.589 to 0.975**	**0.031**
General anxiety Class 3	**2.030**	**1.374 to 2.998**	**<0.001**	**1.843**	**1.190 to 2.945**	**0.002**
Sex	---	---	---	1.205	0.981 to 1.480	0.075
Gestational age	---	---	---	1.016	0.956 to 1.080	0.610
FAI total score	---	---	---	0.988	0.964 to 1.012	0.318
Ethnicity	---	---	---	1.426	0.755 to 2.692	0.274
Maternal age when birth	---	---	---	0.997	0.974 to 1.020	0.784
Cannabis use ever at 15 years	---	---	---	**1.985**	**1.468 to 2.731**	**<0.001**

*Psychotic episode at 24 years, psychotic symptoms ever, not attributable to sleep or fever, and very distressing or having a very negative impact on their social or occupational functioning

Table S7. Logistic regression analyses between 3-classes model of general anxiety and depression at 24

**Moderate depressive disorder at 24 years**						
	**Unadjusted model**			**Adjusted model**		
	OR	CI 95%	p	OR	CI 95%	p
Anxiety Class1 (ref)	---	---	<0.001	---	---	<0.001
Anxiety Class2	0.96	0.79 to 1.16	0.674	1.06	0.86 to 1.32	0.575
Anxiety Class3	**1.83**	**1.37 to 2.45**	**<0.001**	**1.99**	**1.44 to 2.76**	**<0.001**
Sex	---	---	---	**0.60**	**0.50 to 0.73**	**<0.001**
Gestational age	---	---	---	0.98	0.93 to 1.03	0.362
FAI total score	---	---	---	**1.06**	**1.04 to 1.08**	**<0.001**
Ethnicity	---	---	---	0.46	0.20 to 1.06	0.068
Maternal age birth	---	---	---	0.99	0.98 to 1.02	0.654
Cannabis use ever at 15 years	---	---	---	0.84	0.68 to 1.04	0.112

Table S8. Logistic regressions between 3-classes model of general anxiety and anxiety at 24y

Social phobia at 24 years						
	**Unadjusted model**			**Adjusted model**		
	OR	CI 95%	p	OR	CI 95%	p
Anxiety Class1 (ref)	---	---	0.956	---	---	0.987
Anxiety Class2	0.84	0.27 to 2.61	0.764	0.91	0.27 to 3.01	0.874
Anxiety Class3	0.00	0.00 to 0.00	0.993	0.00	0.00 to 0.00	0.993
Sex	---	---	---	0.53	0.18 to 1.56	0.249
Gestational age	---	---	---	1.03	0.75 to 1.42	0.854
FAI total score	---	---	---	**1.18**	**1.10 to 1.28**	**<0.001**
Ethnicity	---	---	---	0.00	0.00 to 0.00	0.996
Maternal age birth	---	---	---	1.00	0.90 to 1.11	0.971
Cannabis use ever at 15 years	---	---	---	0.98	0.35 to 4.01	0.901

Specific phobia at 24 years						
	**Unadjusted model**			**Adjusted model**		
	OR	CI 95%	p	OR	CI 95%	p
Anxiety Class1 (ref)	---	---	0.819	---	---	0.680
Anxiety Class2	1.41	0.49 to 4.09	0.527	1.62	0.55 to 4.77	0.380
Anxiety Class3	0.00	0.00 to 0.00	0.994	0.00	0.00 to 0.00	0.993
Sex	---	---	---	0.00	0.00 to 0.00	0.979
Gestational age	---	---	---	1.14	0.80 to 1.62	0.465
FAI total score	---	---	---	0.98	0.86 to 1.12	0.771
Ethnicity	---	---	---	**7.86**	**1.81 to 34.17**	**0.006**
Maternal age birth	---	---	---	0.93	0.83 to 1.05	0.53
Cannabis use ever at 15 years	---	---	---	0.96	0.31 to 6.32	0.898

Generalized anxiety disorder at 24 years						
**ANXIETY**	**Unadjusted model**			**Adjusted model**		
	OR	CI 95%	p	OR	CI 95%	p
Anxiety Class1 (ref)	---	---	<0.001	---	---	<0.001
Anxiety Class2	1.15	0.97 to 1.37	0.095	**1.34**	**1.11 to 1.62**	**0.003**
Anxiety Class3	**2.21**	**1.70 to 2.85**	**<0.001**	**2.15**	**1.59 to 2.90**	**<0.001**
Sex	---	---	---	**0.51**	**0.43 to 0.61**	**<0.001**
Gestational age	---	---	---	0.97	0.92 to 1.01	0.175
FAI total score	---	---	---	**1.03**	**1.01 to 1.05**	**0.001**
Ethnicity	---	---	---	1.12	0.65 to 1.94	0.681
Maternal age birth	---	---	---	0.99	0.98 to 1.01	0.506
Cannabis use ever at 15 years	---	---	---	**0.68**	**0.56 to 0.82**	**<0.001**

Table S9. Logistic regression analyses between 3-classes model of general anxiety and hypomania at 24 years

Hypomania at 21 years						
**ANXIETY**	**Unadjusted model**			**Adjusted model**		
	OR	CI 95%	p	OR	CI 95%	p
Anxiety Class1 (ref)	---	---	0.341	---	---	0.333
Anxiety Class2	1.04	0.93 to 1.16	0.502	0.87	0.56 to 1.34	0.520
Anxiety Class3	1.18	0.93 to 1.48	0.168	1.49	0.78 to 2.84	0.222
Sex	---	---	---	0.85	0.60 to 1.20	0.358
Gestational age	---	---	---	**1.17**	**1.04 to 1.32**	**0.007**
FAI total score	---	---	---	0.99	0.96 to 1.04	0.965
Ethnicity	---	---	---	2.11	0.86 to 5.17	0.102
Maternal age birth	---	---	---	1.00	0.96 to 1.04	0.940
Cannabis use ever at 15 years	---	---	---	1.11	0.72 to 1.73	0.631

Table S10. Logistic regression analyses between 3-classes model of general anxiety and substance abuse at 24 years

**Alcohol dependence at 24 years**						
**ANXIETY**	**Unadjusted model**			**Adjusted model**		
	OR	CI 95%	p	OR	CI 95%	p
Anxiety Class1 (ref)	---	---	0.029	---	---	0.059
Anxiety Class2	1.22	1.01 to 1.49	0.041	1.18	0.95 to 1.48	0.138
Anxiety Class3	0.71	0.45 to 1.13	0.152	0.62	0.35 to 1.08	0.094
Sex	---	---	---	**1.53**	**1.24 to 1.87**	**<0.001**
Gestational age	---	---	---	**1.10**	**1.03 to 1.17**	**0.005**
FAI total score	---	---	---	**1.02**	**1.00 to 1.04**	**0.034**
Ethnicity	---	---	---	0.34	0.10 to 1.18	0.089
Maternal age birth	---	---	---	1.02	0.99 to 1.04	0.095
Cannabis use ever at 15 years	---	---	---	**1.52**	**1.22 to 2.32**	**<0.001**

**Alcohol abuse at 24 years**						
**ANXIETY**	**Unadjusted model**			**Adjusted model**		
	OR	CI 95%	p	OR	CI 95%	p
Anxiety Class1 (ref)	---	---	0.867	---	---	0.809
Anxiety Class2	0.98	0.84 to 1.15	0.811	1.01	0.84 to 1.21	0.913
Anxiety Class3	0.92	0.68 to 1.25	0.613	0.90	0.64 to 1.26	0.535
Sex	---	---	---	**2.71**	**2.30 to 3.19**	**<0.001**
Gestational age	---	---	---	1.01	0.97 to 1.06	0.526
FAI total score	---	---	---	1.01	0.99 to 1.03	0.229
Ethnicity	---	---	---	1.22	0.74 to 2.01	0.442
Maternal age birth	---	---	---	**0.98**	**0.97 to 0.99**	**0.040**
Cannabis use ever at 15 years	---	---	---	**1.68**	**1.46 to 2.51**	**<0.001**

**Cannabis use at 24 years**						
**ANXIETY**	**Unadjusted model**			**Adjusted model**		
	OR	CI 95%	p	OR	CI 95%	p
Anxiety Class1 (ref)	---	---	0.444	---	---	0.672
Anxiety Class2	0.67	0.36 to 1.24	0.203	0.66	0.27 to 1.63	0.372
Anxiety Class3	0.00	0.00 to 0.00	0.996	0.00	0.00 to 0.00	0.996
Sex	---	---	---	**3.91**	**1.41 to 4.25**	**0.003**
Gestational age	---	---	---	0.97	0.81 to 1.17	0.772
FAI total score	---	---	---	**1.08**	**1.02 to 1.14**	**0.007**
Ethnicity	---	---	---	0.00	0.00 to 0.00	0.997
Maternal age birth	---	---	---	**0.88**	**0.82 to 0.94**	**<0.001**
Cannabis use ever at 15 years	---	---	---	**1.46**	**1.23 to 2.91**	**<0.001**

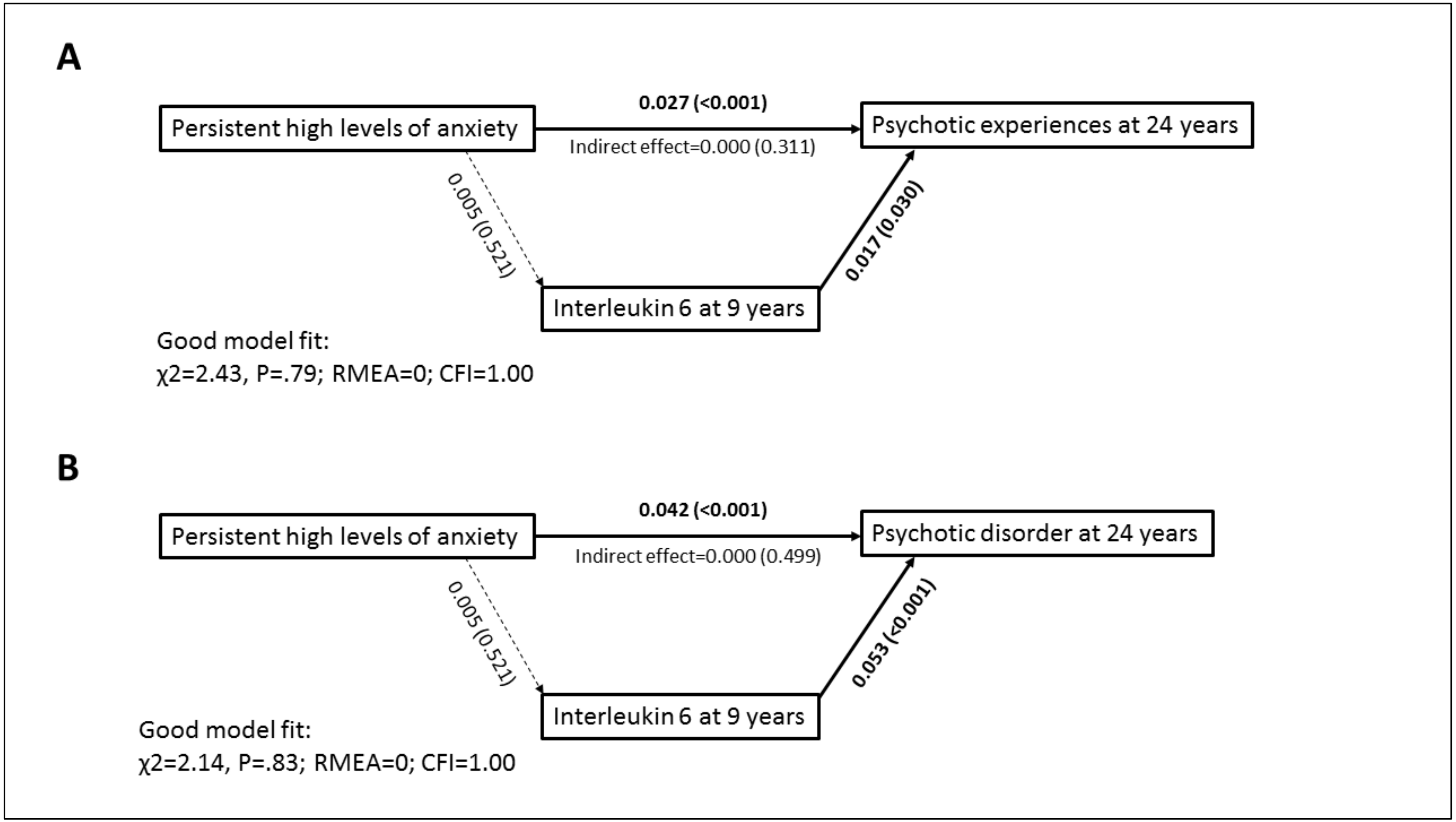

Fig S1. Path diagram showing the main direct associations between persistent anxiety, IL-6 at 9 years and psychotic outcomes at 24 years. This figure shows that IL-6 at years is not a mediator in any of the associations between persistent anxiety and PEs at 24 years or between persistent anxiety and meeting criteria of psychotic disorder at 24 years.

Table S11. Descriptive values (Mean and SD) of the inflammatory marker values in persistent anxiety, PE at 24 years and PD at 24 years

	Persistent anxiety at 8, 10 and 13 years				Psychotic experiences at 24 years				Criteria of psychotic disorder at 24 years			
	Yes		No		Yes		No		Yes		No	
	Mean	SD	Mean	SD	Mean	SD	Mean	SD	Mean	SD	Mean	SD
CRP at 9 years	0.94	3.40	0.79	2.69	0.85	1.53	0.76	2.57	0.93	1.93	0.76	2.55
CRP at 15 years	1.26	3.50	1.24	3.80	1.39	2.26	1.16	3.57	1.25	2.79	1.16	3.54
IL-6 at 9 years	1.33	1.43	1.29	1.59	1.40	1.45	1.29	1.57	1.92	1.78	1.27	1.57
